# Supplementary material for: Mechanisms governing the pioneering and redistribution capabilities of the non-classical pioneer PU.1
Source: Nat Commun. 2020 Jan 21;11:402. doi: 10.1038/s41467-019-13960-2 (PMC6972792; doi:10.1038/s41467-019-13960-2)
Supplement: Supplementary file 7 — Source data [file 41467_2019_13960_MOESM7_ESM.zip › Source_Data/FigureS2/FigureS2B_MotifScanOutput/KmeansCluster12/homerResults/motif8.similar.html]

motif8

## Information for motif8

A
G
T
C
G
A
C
T
A
C
G
T
A
G
T
C
A
G
T
C
A
C
G
T
A
C
G
T
A
T
G
C
T
G
A
C
G
C
A
T
T
C
A
G
  
Reverse Opposite:  

A
G
T
C
C
G
T
A
A
C
T
G
T
A
C
G
T
G
C
A
T
G
C
A
T
C
A
G
T
C
A
G
T
G
C
A
C
T
G
A
T
C
A
G
  

|  |  |
| --- | --- |
| p-value: | 1e-41 |
| log p-value: | -9.482e+01 |
| Information Content per bp: | 1.689 |
| Number of Target Sequences with motif | 348.0 |
| Percentage of Target Sequences with motif | 19.37% |
| Number of Background Sequences with motif | 4045.9 |
| Percentage of Background Sequences with motif | 8.79% |
| Average Position of motif in Targets | 102.7 +/- 57.4bp |
| Average Position of motif in Background | 100.4 +/- 69.3bp |
| Strand Bias (log2 ratio + to - strand density) | -0.1 |
| Multiplicity (# of sites on avg that occur together) | 1.04 |
| Motif File: | file (matrix) reverse opposite |

### Similar de novo motifs found

|  |  |  |  |  |  |  |  |
| --- | --- | --- | --- | --- | --- | --- | --- |
| Rank | Match Score | Redundant Motif | P-value | log P-value | % of Targets | % of Background | Motif file |
| 1 | 0.844 | T G A C G T C A A T G C G A C T A G C T G T A C A G T C G A C T A C G T A T G C G A T C A C G T | 1e-29 | -67.087272 | 6.84% | 1.96% | motif file (matrix) |
| 2 | 0.772 | G C T A C T A G A T C G C T G A C T G A C T A G A T C G T C G A C T G A T C A G A C G T C A T G A C T G | 1e-28 | -65.946912 | 8.79% | 3.07% | motif file (matrix) |
